# Dendritic Gold Nanoparticles Loaded on 3D Graphene-like Surface and Layer-by-Layer Assembly for Enhanced Glucose Biosensing

**DOI:** 10.3390/bios15040246

**Published:** 2025-04-12

**Authors:** Zifeng Zhu, Yiming Zhao, Yongming Ruan, Xuexiang Weng, Gesmi Milcovich

**Affiliations:** 1 College of Chemistry and Materials Science, Key Laboratory of the Ministry of Education for Advanced Catalysis Materials, Zhejiang Normal University, Jinhua 321004, China; 15356970090@zjnu.edu.cn (Z.Z.); 2080513985@zjnu.edu.cn (Y.Z.); 2 College of Life Sciences, Zhejiang Normal University, Jinhua 321004, China; ruanym@zjnu.cn; 3 Department of Life Sciences, University of Modena and Reggio Emilia, 41124 Modena, Italy

**Keywords:** dendritic nanogold, self-assembly, chitosan, glucose biosensor, diabetes mellitus

## Abstract

Background/Objectives: In this study, AuDNs/EPLE composite electrodes with hierarchical dendritic nanogold structures were fabricated using the in situ electrodeposition of gold nanoparticles through the *i*-*t* method. Methods: A conductive polymer composite membrane, PEDOT, was synthesized via the electropolymerization of EDOT and the negatively charged PSS^−^. The negatively charged SO_3_^−^ groups on the surface of the PEDOT membrane were electrostatically adsorbed with the glucose oxidase (GOD) enzyme and a positively charged chitosan co-solution (GOD/chit^+^). Using a layer-by-layer self-assembly approach, GOD was incorporated into the multilayers of the composite electrode to create the composite GOD/chit^+^/PEDOT/AuDNs/EPLE. Results: Electrochemical analysis revealed a GOD surface coverage of 8.5 × 10^−10^ mol cm^−2^ and an electron transfer rate of 1.394 ± 0.02 s^−1^. The composite electrode exhibited a linear response to glucose in the concentration range of 6.923 × 10^−2^ mM to 1.54 mM, with an apparent Michaelis constant of 0.352 ± 0.02 mM. Furthermore, the GOD/chit^+^/PEDOT/AuDNs/EPLE also showed good accuracy of glucose determination in human serum samples. Conclusions: These findings highlight the potential of the GOD/chit^+^/PEDOT/AuDNs/EPLE composite electrode in the development of efficient enzymatic biofuel cells for glucose sensing and energy harvesting applications.

## 1. Introduction

Diabetes mellitus is a chronic metabolic disorder characterized by hyperglycemia, which can lead to severe complications if not carefully managed and targeted [1]. Accurate, rapid, and cost-effective blood glucose detection is pivotal for effective disease management and overall well-being. The measurement of blood glucose concentration is still the primary diagnostic parameter for diabetes, and advancements in glucose monitoring technologies continue to play a key role in the improvement of patient outcomes. A key advancement was the introduction of the ExacTech meter (MediSense, now Abbott) in 1987, the first electrochemical glucose biosensor for home use [2]. This pioneering fixed-volume device provided a highly convenient and accessible method for both home and clinical blood glucose monitoring.

On the other hand, the following decades demonstrated an evolving development in sensor-based glucose quantification, mainly in optical [3,4] and electrochemical biosensing [5,6,7,8,9]. Electrochemical sensors have gathered remarkable attention due to their high sensitivity, low detection limits, ease of use, and potential for in situ continuous monitoring, which can further enhance diabetes management beyond the capabilities of the fixed-volume device.

Currently, most electrochemical glucose sensors rely on either enzymatic or non-enzymatic methods [10,11,12,13]. Hence, glucose oxidase (GOD) is widely employed, as it belongs to the class of enzymes associated with glucose metabolism. Therefore, it represents a key feature, due to its fast mechanism of action, high stability, and specificity [13,14]. However, the enzymatic approach is often hindered by the relatively slow electron transfer (ET) rate between the active site of GOD and the electrode surface. This phenomenon is primarily due to the deep positioning of the flavin adenine dinucleotide (FAD) redox center within the enzyme structure. To enhance DET efficiency, several strategies have been explored, including the use of nanomaterials and advanced immobilization techniques.

Carbon-based materials, such as graphene [15] and carbon nanotubes [16], offer large surface areas and excellent electrical conductivity, thus enhancing electron transfer between an enzyme and an electrode. Metal nanoparticles [17,18], mainly gold nanoparticles (AuNPs), can further promote the catalytic activity and biocompatibility of electrodes. On the other hand, the method of enzyme immobilization has a key role while optimizing electron transfer between an enzyme and an electrode. In particular, the general enzyme immobilization techniques, such as embedding, crosslinking, physical adsorption, and layer-by-layer (LBL) self-assembly, are essential for the development of enzyme-linked electrochemical sensors. These methods each present unique advantages and limitations [19]. In detail, the LBL assembly approach is particularly advantageous, as it allows for precise control over the interface distance and the conformation of the enzyme, leading to enhanced stability and catalytic performance [20]. Conductive polymers, such as poly (3,4-ethylenedioxythiophene) (PEDOT), are frequently utilized in LBL assembly due to their ability to delocalize charge carriers, thus promoting electron transfer between the enzyme’s active site and the electrode. PEDOT presents remarkable electrochemical stability, a low-energy band gap, and biocompatibility. Hence, it is often polymerized in combination with poly (4-styrenesulfonic acid) (PSS), a polyelectrolyte that improves water solubility and dispersion. This combination results in composite films with high conductivity and solubility, therefore suitable for enzyme immobilization in electrochemical sensors [20,21,22].

In our previous work, we developed a 3D graphene-like electrode (EPLE) through electrochemical pretreatment of a pencil lead electrode [23]. This modified electrode exhibited enhanced electrochemical properties and was further functionalized with nanomaterials, such as gold nanoparticles and copper oxide, for non-enzymatic glucose sensing [24].

Based on our previous optimization research, this study aims to develop a highly sensitive and selective glucose biosensor by combining the advantages of 3D graphene-like electrodes, dendritic gold nanoparticles (AuDNs), and a layer-by-layer assembly approach. By immobilizing GOD onto a multilayer film of chitosan and PEDOT on the AuDNs-modified EPLE surface (GOD/chit^+^/PEDOT/AuDNs/EPLE), we hypothesize that it is possible to enhance glucose DET efficiency and improve the overall performance of the biosensor.

## 2. Materials and Methods

### 2.1. Reagents and Materials

The pencil lead electrode (PLE, 2B, 0.5 mm in diameter) was obtained from Ningbo Friendly Stationery Co., Ltd., Ningbo, China. Chitosan (chit^+^, low molecular weight, with a deacetylation degree of 85%, exhibiting high positive charge density), sodium sulfate (Na_2_SO_4_), sodium dihydrogen phosphate, glucose, potassium ferricyanide (K_3_[Fe(CN)_6_]), potassium ferrocyanide (K_4_[Fe(CN)_6_]), potassium chloride (KCl), glacial acetic acid, and chlorauric acid (HAuCl_4_·4H_2_O) were sourced from China Pharmaceutical Group Chemicals Co., Ltd. (Tianjin, China). The 3,4-ethylenedioxythiophene (EDOT) solution was purchased from Mirida Technology Co., Ltd., Green Valley, CA, USA. Poly (sodium styrene sulfonate) (NaPSS) and glucose oxidase (GOD) were sourced from (17.3 U mg^−1^) Sigma-Aldrich (St. Louis, MA, USA). The GOD solution was prepared by dissolving glucose oxidase powder (5 mg mL^−1^) in Tris-HCl buffer (pH 8.9, 0.05 mol L^−1^).

### 2.2. Instrumentation

Scanning electron microscope (SEM) images of the electrode surface were obtained using a Hitachi S-4800 scanning electron microscope (Hitachi, Tokyo, Japan). An X-ray photoelectron spectroscopy (XPS) analysis of the electrode surface was performed using a K-Alpha XPS spectrometer (ESCALAB 250, Thermo Fisher Scientific, Waltham, MA, USA) with an Al Kα X-ray radiation source (1486.6 eV). The X-ray diffraction (XRD) spectra of the electrode surface were characterized using a Bruker D8-ADVANCE diffractometer (Bruker Co., Bremen, Germany) with Cu Kα radiation (λ = 1.54056 Å).

For electrochemical measurements, a conventional three-electrode system was employed, with a GOD/chit^+^/PEDOT/AuDNs/EPLE as the working electrode, a platinum wire as the counter electrode, and a saturated calomel electrode (SCE) as the reference electrode. A phosphate-buffered saline (PBS) solution (0.2 M, pH = 7.19) was used as the electrolyte.

### 2.3. Preparation of AuDNs/EPLE and Its Electrochemical Active Area Test

For the deposition of AuDNs, the EPLE electrodes were immersed in HAuCl_4_ solutions at concentrations of 1.27, 1.85, 3.44, and 6.03 mM and electrodeposited under different potentials (−0.5 V, −0.3 V, −0.1 V, 0.1 V, and 0.3 V) for different durations (600, 1200, 3600, 5400, and 7200 s). Following deposition, the AuDNs/EPLE electrodes were thoroughly rinsed with deionized water and dried at RT. The electrochemical active surface area (ECSA) of the AuDNs/EPLEs was assessed via cyclic voltammetry (CV) in 0.5 M H_2_SO_4_. The ECSA was calculated using the following equation [25]:

(1) $$\mathrm{ECSA}\;=\frac{Q_{0}}{Q_{0s}}(Q_{0}=\frac{S}{V})$$

where *Q*_0_ represents the charge required for oxygen adsorption, *Q*_os_ (0.386 mC cm^−2^) is the charge density required to reduce monolayer oxygen, *S* is the integrated area of the cathode reduction peak, and *V* is the scan rate during the CV test (100 mV s^−1^).

### 2.4. Preparation of GOD/chit^+^/PEDOT/AuDNs/EPLE

The fabrication of the enzymatic electrode involved a two-step process: electropolymerization of EDOT onto the electrode surface and subsequent enzyme immobilization.

First, a polymerization solution containing 0.15 mM EDOT and 1.5 mM NaPSS was prepared by stirring and heating at 60 °C. The AuDNs/EPLE was then immersed in this solution at room temperature, and electropolymerization was carried out using cyclic voltammetry (CV) over a potential range of −0.6 to 0.8 V at a scan rate of 50 mV s^−1^. After 10 CV cycles, a light grey, porous PEDOT:PSS film was formed on the electrode surface. The presence of negatively charged SO_3_^−^ groups in the film enhanced the following deposition of a positively charged enzyme layer.

For the enzyme immobilization, a glucose oxidase (GOD) and chitosan co-solution (GOD/chit^+^) was prepared by mixing a 1% (*w*/*v*) chitosan solution (in acid conditions, with 1% acetic acid) with a GOD solution (5 mg mL^−1^) in equal volumes. The mixture was homogenized using a vortex mixer for 30 s. Chitosan, a naturally derived biocompatible polymer, is widely utilized in biomedical applications due to its unique physicochemical properties [26]. With a p*K*_a_ of approximately 6.3, chitosan remains positively charged in acidic solutions, promoting electrostatic interactions with the negatively charged PSS in PEDOT:PSS under ambient conditions [27]. Furthermore, its hydrophilic backbone provides a favorable microenvironment for entrapped enzymes, preserving their bioactivity and stability in both dry and wet states.

The PEDOT-modified electrode was then immersed in the GOD/chit^+^ co-solution for 20 min, followed by rinsing with water and drying under a nitrogen stream. This process of electropolymerization and enzyme entrapment was repeated until three GOD layers were sequentially deposited onto the electrode surface, leading to the final GOD/chit^+^/PEDOT/AuDNs/EPLE composite electrode.

### 2.5. Electrochemical Characterization of Enzyme Electrodes

The electrochemical properties of the enzyme-modified electrodes were evaluated through cyclic voltammetry (CV). The number of electrons transferred (*n*) during the redox process was calculated from the integration of the reduction peak current at a scan rate of 100 mV s^−1^ according to the following equation:

(2) $$i_{p}=\mathrm{nFQ}\upsilon /4\mathrm{RT}$$

where *i*_p_ is the peak current (A), *F* is the Faraday constant (96,485 C mol^−1^), *Q* is the charge (C), *υ* is the scan rate (V s^−1^), *R* is the universal gas constant (8.314 J mol^−1^ K^−1^), and *T* is the absolute temperature (K).

The surface coverage (*Γ*, mol cm^−2^) of the enzyme on the electrode surface was determined by integrating the reduction peak current at 100 mV s^−1^, using the following equation [28]:

(3) Q=ΓnFA

where *A* is the electrode surface area.

The electron transfer rate constant (*k*_s_) and electron transfer coefficient (α) of the GOD/chit^+^/PEDOT/AuDNs/EPLE were determined using Laviron’s theory [28], which describes the relationship between peak-to-peak separation (Δ*E*_p_) and the electron transfer kinetics:

(4) $$\Delta E_{p}=\frac{2.3\mathrm{RT}}{n}F\alpha \left(1-\alpha \right)[\alpha \log\left(1-\alpha \right)+(1-\alpha )\log\alpha -\log\left(\frac{\mathrm{RT}}{\mathrm{nFv}}\right)-\log k_{s}]$$

(5) $$E_{\mathrm{pa}}=E^{\prime 0}-(\frac{\mathrm{RT}}{\alpha \mathrm{nF}})\ln\left(\frac{\alpha \mathrm{nFv}}{\mathrm{RT}k_{s}}\right)$$

(6) $$E_{\mathrm{pc}}=E^{\prime 0}+[\frac{\mathrm{RT}}{\left(1-\alpha \right)\mathrm{nF}}]\ln\left[\frac{(1-\alpha )\mathrm{nFv}}{\mathrm{RT}k_{s}}\right]$$

where *E*_pa_ and *E*_pc_ are the cathodic and anodic peak potentials, respectively. *E*′ is the formal potential of the redox system.

### 2.6. Determination of Real Samples

The human serum samples were used in accordance with the guidelines of the Declaration of Helsinki, and their use was approved by the Medical Ethics Review Committee of Jinhua Municipal Central Hospital (Approval No. 2025-71, dated 28 February 2025). The samples were analyzed using the *i*-*t* method with the standard addition method. The measurements were performed at a potential of −0.45 V in 10 mL of 0.2 M PBS (pH 7.19) under stirring. A volume of 200 μL of blood serum was injected into the buffer solution. The resulting current was substituted into the calibration curve to calculate the corresponding glucose concentrations. The measurements were performed in triplicate for each sample.

## 3. Results and Discussion

### 3.1. Optimization of AuDNs Deposition Conditions on EPLE

**Figure 1:**
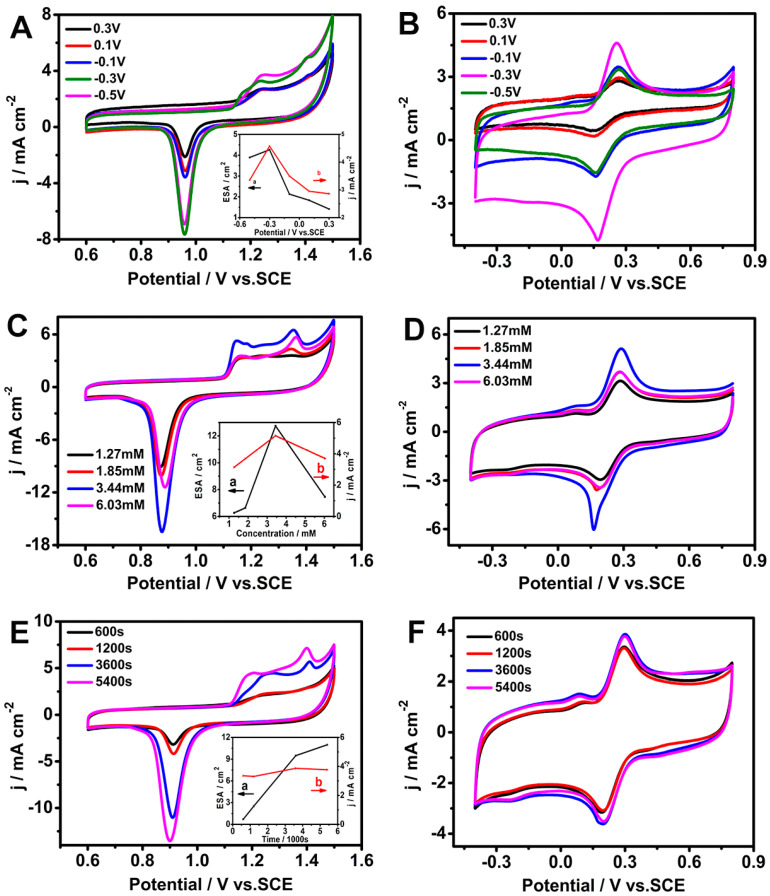
Electrochemical characterization of AuDNs/EPLEs prepared under different conditions. (**A**,**C**,**E**) Cyclic voltammograms (CVs) of AuDNs/EPLEs in 0.5 M H_2_SO_4_. Insets in (**A**,**B**) show the corresponding ECSA and current density, respectively. (**B**,**D**,**F**) CVs of AuDNs/EPLEs in 0.1 M KCl solution containing 5 mM [Fe(CN)_6_]^3−^/[Fe(CN)_6_]^4−^.

To optimize the synergistic effect of AuDNs and EPLEs, the deposition potential, electrolyte concentration, and deposition time were investigated. Figure 1A depicts the cyclic voltammograms (CVs) of the AuDNs/EPLE at different potentials in a 0.5 M H_2_SO_4_ solution. The corresponding electrochemical active surface area (ECSA) was calculated for each deposition potential (Figure 1A inset). The results revealed that the electrode deposited at −0.3 V exhibited the highest ECSA. Additionally, this deposition potential led to the highest current density with 5 mM [Fe(CN)_6_]^3−^ (Figure 1B).

The concentration of HAuCl_4_ remarkably influenced the electrochemical performance of the AuDNs/EPLE too. As shown in Figure 1C,D, increasing the HAuCl_4_ concentration from 1.27 mM to 3.44 mM resulted in a progressive increase in the ECSA and the corresponding current density with 5 mM [Fe(CN)_6_]^3−^. However, a further increase in concentration led to a decrease in both the ECSA and current density.

Under the optimized conditions of −0.3 V deposition potential and 3.44 mM HAuCl_4_ concentration, the influence of deposition time on the electrochemical properties of the AuDNs/EPLE was investigated. Figure 1E demonstrates that the ECSA increased with increasing deposition time. Indeed, Figure 1F shows that the highest current density was achieved at a deposition time of 3600 s. Considering the reagent and time efficiency, 3600 s was chosen as the optimal deposition time for subsequent experiments.

These optimizations allowed us to reach a balance between maximizing the surface area of the AuDNs for enhanced electron transfer and maintaining a well-defined morphology for optimal catalytic activity.

**Figure 2:**
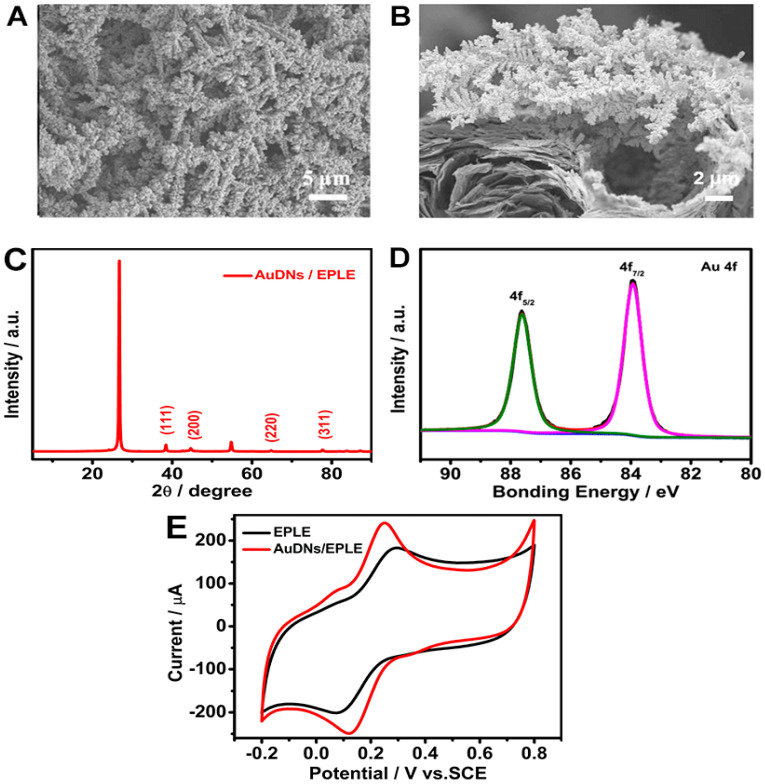
Characterization of AuDNs/EPLEs: (**A**) top view and (**B**) cross-sectional SEM images of AuDNs/EPLE; (**C**) XRD pattern of AuDNs/EPLE; (**D**) high-resolution XPS spectrum of Au 4f for AuDNs/EPLE; (**E**) cyclic voltammograms of EPLE and AuDNs/EPLE in 0.1 M KCl solution containing 5 mM [Fe(CN)_6_]^3−^/[Fe(CN)_6_]^4−^.

The SEM images in Figure 2A,B reveal a well-defined dendritic structure, with Au uniformly distributed on the graphene-like EPLE surface. The XRD pattern in Figure 2C confirms the presence of metallic gold, with characteristic peaks related to the (111), (200), (220), and (311) crystal planes [29], while the diffraction peak at 2θ = 26.5° and 54.7° corresponded to the (002) and (004) graphite hexagonal structure. The high-resolution XPS spectrum in Figure 2D further confirms the presence of metallic gold, with binding energies at 83.91 eV and 87.63 eV for Au 4f_7/2_ and Au 4f_5/2_, respectively [30].

The electrochemical performance of the AuDNs/EPLE was evaluated by cyclic voltammetry in a solution containing 5 mM [Fe(CN)_6_]^3−^/[Fe(CN)_6_]^4−^. As shown in Figure 2E, the AuDNs/EPLE exhibited a significantly higher current density and a narrower Δ*E*_p_ compared to the bare EPLE. This enhanced electrochemical performance can be associated with the increased surface area and improved electron transfer kinetics provided by the dendritic gold nanoparticles.

### 3.2. Electrochemical Characterization of GOD/chit^+^/PEDOT/AuDNs/EPLE

**Figure 3:**
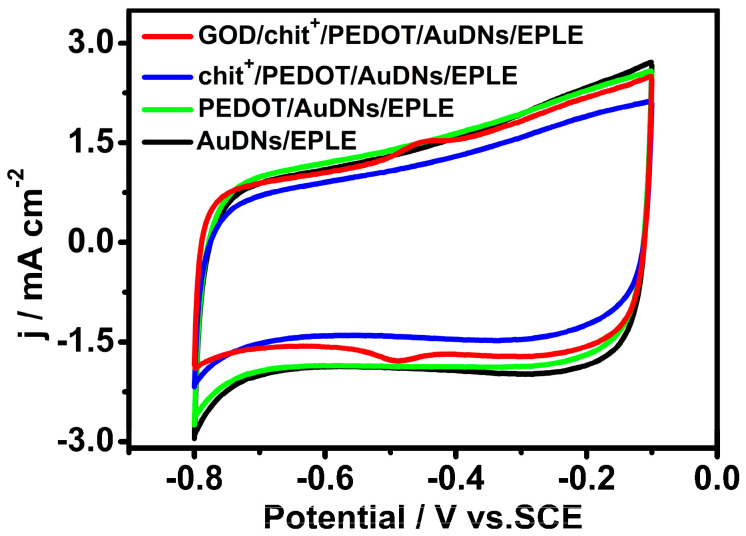
CV curves of different electrodes in air-saturated PBS (pH = 7.19, 0.1 M) buffer solution, scanning rate 50 mV s^−1^.

To investigate the electrochemical behavior of the immobilized GOD, cyclic voltammetry (CV) was performed in a pH 7.19 phosphate buffer solution. Figure 3 shows the CV curves of different electrodes. Interestingly, just the GOD/chit^+^/PEDOT/AuDNs/EPLE exhibited a pair of well-defined redox peaks at −0.448 V and −0.488 V, referring to the redox reaction of the FAD cofactor in GOD. These results indicate that the layer-by-layer assembly strategy effectively immobilized GOD onto the electrode surface and promoted direct electron transfer (DET) between the enzyme and the electrode.

**Figure 4:**
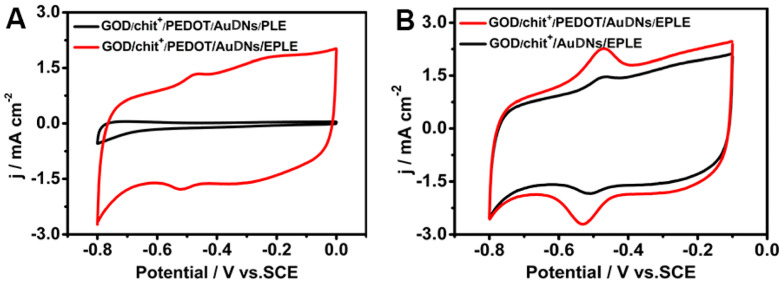
CV curves of GOD on different electrodes: (**A**) before electrochemical oxidation (PLE) and after (EPLE); (**B**) before and after modification of the conductive polymer, the buffer solution is PBS with pH = 7.19, scanning rate 50 mV s^−1^.

To further demonstrate the role of the 3D graphene-like structure and conductive polymer in enhancing enzyme electrochemistry, comparative studies were performed. Figure 4A shows that no significant redox peaks were observed for GOD immobilized on a bare PLE electrode, highlighting the importance of the 3D graphene structure for efficient enzyme immobilization and electron transfer. Figure 4B indicates the significant enhancement in the current density of the GOD/chit^+^/PEDOT/AuDNs/EPLE, compared to the electrode without PEDOT modification. This confirms that the conductive polymer layer plays a key role in promoting electron transfer between the enzyme and the electrode.

### 3.3. Kinetic Parameters of GOD/chit^+^/PEDOT/AuDNs/EPLE

**Figure 5:**
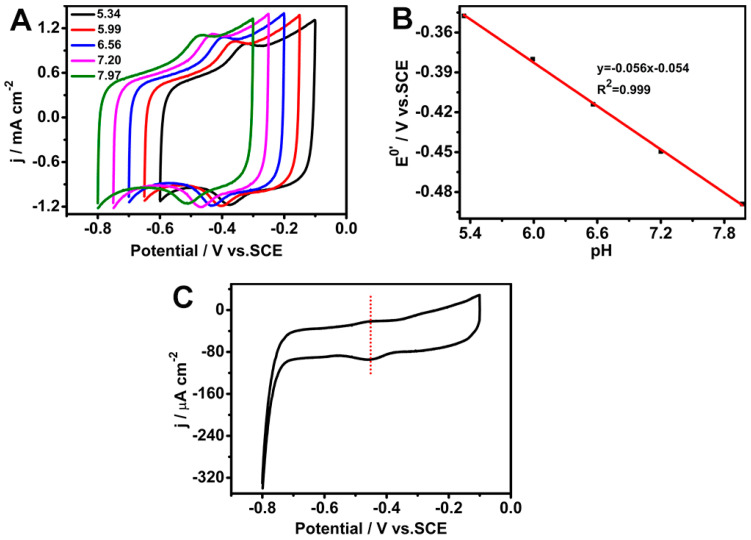
Electrochemical characterization of the GOD/chit^+^/PEDOT/AuDNs/EPLE: (**A**) CVs at pH 5.34, 5.99, 6.56, 7.20, and 7.97, scan rate 50 mV s^−1^; (**B**) plot of the peak potential versus pH; (**C**) CV curve at a scan rate of 1 mV s^−1^.

Figure 5A shows the CV curves of the GOD/chit^+^/PEDOT/AuDNs/EPLE bioanode at various pH values. As the pH increased, the redox peak potential shifted negatively, indicating a pH-dependent redox process. The slope of the linear relationship between the peak potential and pH was −56 mV pH^−1^ (*R*^2^ = 0.999) (Figure 5B), which is consistent with the theoretical value for a two-electron, two-proton transfer reaction involving the FAD cofactor of GOD. The CV curve in Figure 5C, recorded at a scan rate of 1 mVs^−1^, exhibits a pair of symmetrical redox peaks with a negligible peak-to-peak separation. This indicates a quasi-reversible electrochemical process, suggesting that the electron transfer between GOD and the electrode is efficient and minimally hindered.

(7) $$\mathrm{GOD}\left(\mathrm{FAD}\right)+2e^{-}+2H^{+}↔\mathrm{GOD}\left({\mathrm{FADH}}_{2}\right)$$

**Figure 6:**
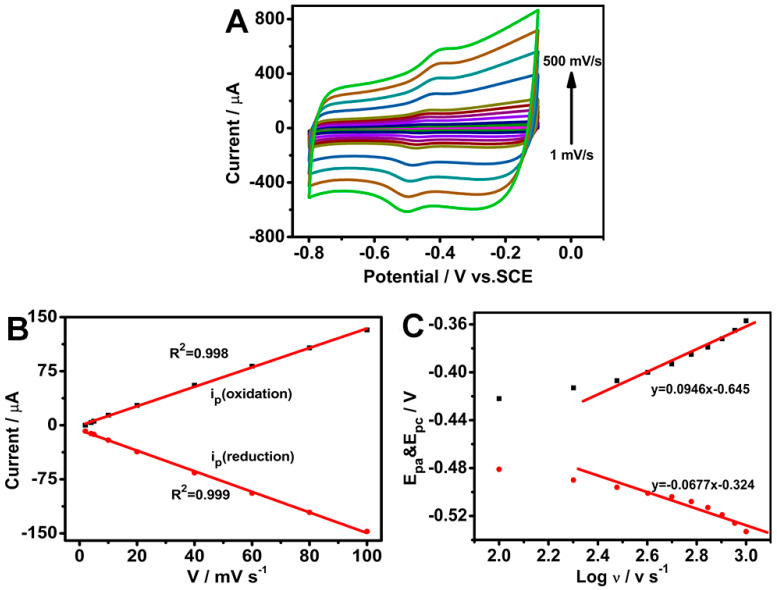
Electrochemical characterization of the GOD/chit^+^/PEDOT/AuDNs/EPLE: (**A**) CVs at different scan rates (1, 10, 20, 40, 60, 80, 100, 200, 300, 400 and 500 mV s^−1^) in pH 7.19 PBS buffer; (**B**) plot of the peak current versus scan rate; (**C**) plot of the peak potential versus the logarithm of the scan rate.

CV was performed at different scan rates to investigate the electrochemical kinetics of the GOD/chit^+^/PEDOT/AuDNs/EPLE. As shown in Figure 6A,B, the peak currents (both anodic and cathodic) increased linearly with the scan rate, indicating a surface-controlled process. The calculated electron transfer number (*n*) from the peak current and charge integration was approximately 2 (Equation (2)), consistent with the two-electron transfer process involved in the redox reaction of the FAD cofactor in GOD. Additionally, the surface coverage (*Γ*) of GOD on the electrode surface was determined to be 8.5 × 10^−10^ mol cm^−2^ (Equation (3)) [31].

Moreover, as per Figure 6C, the peak-to-peak separation (Δ*E*_p_) increased with an increasing scan rate (greater than 100 mV s^−1^), suggesting a quasi-reversible process. The linear relationship between the peak potentials (*E*_pa_ and *E*_pc_) and the logarithm of the scan rate (log *v*) was used to define the electron transfer coefficient (α) (Equations (5) and (6)) and the rate constant (*k*_s_) (Equation (4)). The calculated α value was 0.583, and the *k*_s_ value was 1.394 ± 0.02 s^−1^. These results indicate that the composite electrode exhibits efficient electron transfer kinetics and good electrochemical performance.

### 3.4. Michaelis Constant of GOD/chit^+^/PEDOT/AuDNs/EPLE

**Figure 7:**
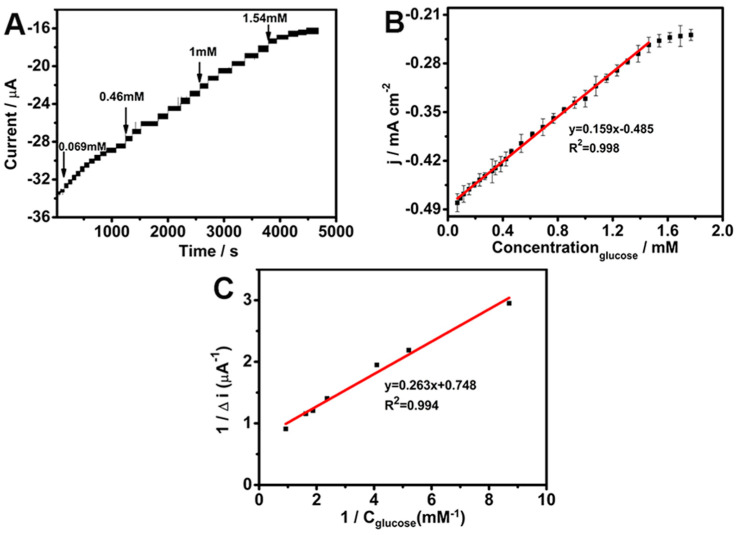
(**A**) Amperometric response of GOD/chit^+^/PEDOT/AuDNs/EPLE to glucose; (**B**) linear fitting of glucose; (**C**) Michaelis constant curve.

In order to investigate the glucose sensing ability of the biosensor, the amperometric response under −0.45 V of the GOD/chit^+^/PEDOT/AuDNs/EPLE was examined with subsequent additions of glucose to a pH 7.19 PBS (Figure 7A). Equation (8) represents the oxidation of glucose oxidase (GOD), where GOD(FADH_2_) is oxidized in the presence of oxygen, which subsequently catalyzes the oxidation of glucose to gluconolactone (Equation (9)). Equation (8) leads to the simultaneous reduction of oxygen to hydrogen peroxide (H_2_O_2_) [32]. As the glucose concentration increases, the availability of oxygen at the electrode surface decreases, resulting in a decline in the oxygen reduction current. This depletion of oxygen leads to a corresponding reduction in the overall current response at higher glucose concentrations.

(8) $$\mathrm{GOD}\left({\mathrm{FADH}}_{2}\right)+O_{2}\to \mathrm{GOD}\left(\mathrm{FAD}\right)+H_{2}O_{2}$$

(9) $$\mathrm{GOD}\left(\mathrm{FAD}\right)+\mathrm{glucose}\to \mathrm{GOD}\left({\mathrm{FADH}}_{2}\right)+\mathrm{gluconolactone}$$

Figure 7B presents the linear calibration curve for glucose detection, which exhibits a linear range of 6.923 × 10^−2^ mM to 1.54 mM, a sensitivity of 0.159 mA mM^−1^ cm^−2^, and a detection limit of 1.4 × 10^−3^ mM. In addition, Table 1 provides a comparison with previously reported glucose sensors using similar materials. Remarkably, the as-prepared GOD/chit^+^/PEDOT/AuDNs/EPLE sensor shows a low detection limit and high sensitivity toward glucose, exhibiting performance metrics that are comparable to, and in certain instances, surpass, those of previously reported sensors. This superior performance can be attributed to the synergistic integration of the composite electrode. Specifically, the PEDOT and AuDNs/EPLE provide a highly conductive matrix that facilitates efficient electron transfer between the enzyme’s active site and the electrode surface, thereby enhancing the bioelectrode’s electrochemical response. Furthermore, the incorporation of chitosan (chit^+^) enhances enzyme immobilization, maintaining GOD activity over extended periods, which contributes to the improved sensitivity and long-term stability of the bioelectrode.

The Michaelis–Menten constant $\left(k_{m}^{app}\right)$ is a kinetic parameter that reflects the affinity of the enzyme for its substrate [22]. Figure 7C shows the Lineweaver–Burk plot, which was used to determine the $k_{m}^{app}$ value of 0.352 ± 0.02 mM [33].

(10) $$\frac{1}{I_{SS}}=\frac{1}{I_{max}}+\frac{k_{m}^{app}}{I_{max}c}$$

where *I*_SS_ is the instantaneous current of the electrode, *I*_max_ is the maximum response of the electrode, c is the substrate concentration, and $k_{m}^{app}$ is the Michaelis constant. This relatively low *K*_m_ value demonstrates a high affinity of the immobilized GOD for glucose, suggesting efficient enzyme–substrate interactions [33].

**Table 1:** Comparison of the performances of similar materials for glucose sensing.

Sample Source	Electrode	Linear Range (mM)	Detection Limit (μM)	Sensitivity (μA mM^−1^ cm^−2^)	Ref.
Human serum	Au–PEDOT–ERGO ^a^	0.1–100	0.12	696.9	[34]
Human serum	Ni (OH)_2_@PEDOT–rGO ^b^	0.002–7.1	0.6	346	[35]
Beverages	PEDOT/GO_x_	0.01–0.8	165	111.78	[36]
Grape wines	Au/PE DOT/{chit^+^(GO_x_)/PSS^−^/chit^+^(GO_x_)}	0.1–14	41	237	[27]
Fruit juice Human serum or sweat	PEDOT:SCX/MXene/GOX ^c^ CHIT(GO_x_)/AuLr-TiND ^d^	0.5–8.0 0.1–7.52, 7.52–40	0.0225 14.38	/ 13.23, 3.79	[37] [38]
Human serum, urine, and saliva	MPC-CHT-GOx ^e^	0.25–3.0	4.1	56.12	[39]
Human serum	Chit/Au/GOD ^f^	0.005–2.4	2.7	/	[40]
Human blood and urine	GOx/Au–PtNPs/CNTs/CS ^g^	0.001–7.0	0.2	8.53	[41]
Urine	PEC/AuNPs/GOD/Au ^h^	0.01–7.0	5.0	283.9	[42]
Human serum	Nf-GOx/PB/AuNS/GR ^i^	0.025–1.0	88	/	[43]
Human serum	GOD/chit^+^/PEDOT/AuDNs/EPLE	0.069–1.54	1.4	159	This work

^a^ ERGO: electrochemically reduced graphene oxide; ^b^ rGO: reduced graphene oxide; ^c^ SCX: 4-sulfocalix arene; MXene: Ti_3_C_2_T_x_; ^d^ chitosan/GOx immobilized onto laser-processed Au-Ti electrode; ^e^ glucose oxidase (GOx) immobilized on chitosan-supported mesoporous carbon nanocomposite; ^f^ chitosan hydrogel incorporated with GOx and gold nanoparticles; ^g^ GOx immobilized in gold–platinum alloy nanoparticles (Au–PtNPs) electrodeposited on multiwall carbon nanotubes (CNTs) in chitosan film; ^h^ chitosan/kappa-carrageenan doped with gold nanoparticles (AuNPs) encapsulating GOD deposited on a Au electrode; ^i^ graphite rod (GR) electrode modification by gold nanostructures (AuNS) and Prussian blue (PB) with GOx.

### 3.5. Stability Studies of the GOD/chit^+^/PEDOT/AuDNs/EPLE

**Figure 8:**
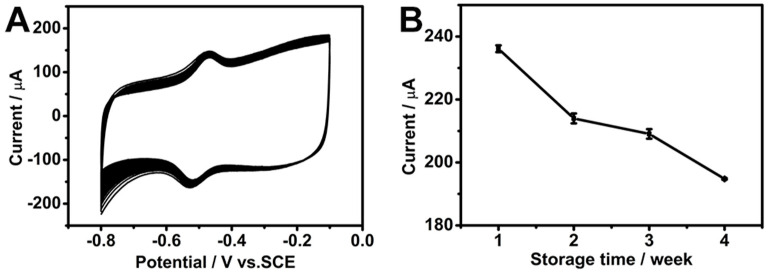
(**A**) The CV curves of the GOD/chit^+^/PEDOT/AuDNs/EPLE in an O_2_-saturated PBS buffer solution (pH = 7.19) scanned for 100 cycles at a scan rate of 50 mV s^−1^. (**B**) The long-term storage stability of the electrode at a scan rate of 200 mV s^−1^.

CV was performed for 100 cycles at a scan rate of 50 mV s^−1^ in a pH 7.19 phosphate buffer solution with saturated O_2_ in order to assess the stability of the composite electrode. As shown in Figure 8A, the electrode retained approximately 90% of its initial peak current after 100 cycles, proving excellent electrochemical stability. Furthermore, the long-term storage stability of the electrode was evaluated by storing the electrode at 4 °C in a refrigerator for one month. After this storage period, it exhibited a retention of 82.5% of its initial current response, indicating good stability over time (Figure 8B).

These results highlight the robust nature of the GOD/chit^+^/PEDOT/AuDNs/EPLE as a promising candidate for practical applications in biosensors and biofuel cells.

### 3.6. Analysis of Real Samples

To further validate the application of the developed electrochemical glucose sensor, the GOD/chit^+^/PEDOT/AuDNs/EPLE biosensor was used to detect glucose concentrations in human serum samples obtained from the hospital. Five serum samples with different glucose concentrations were spiked into 10 mL of the PBS (0.2 M, pH 7.19) solution and analyzed at a working potential of −0.45 V under stirring. As shown in Table 2, the glucose concentrations measured by the biosensor were comparable to the hospital test results. The current measurement procedure, which involves sample dilution and stirring, is more suitable for laboratory settings than home applications. On the other hand, these findings highlight the biosensor’s strong potential for accurate glucose detection in real biological samples, with possible future applications for point-of-care use. 

## 4. Conclusions

In this work, AuDNs/EPLE composite electrodes with hierarchical dendritic nanostructures were prepared through an in-situ electrodeposition of gold nanoparticles by the *i-t* method. Conductive polymer composite membrane PEDOT was obtained by the electropolymerization of conductive polymer EDOT and negatively charged PSS. The negatively charged SO_3_^−^ on the surface of the PEDOT membrane was electrostatically adsorbed with the glucose oxidase (GOD) enzyme and the positively charged chitosan co-solution (GOD/chit^+^); GOD was fixed in the multilayers of the composite electrode to obtain the GOD/chit^+^/PEDOT/AuDNs/EPLE through a layer-by-layer self-assembly method. By studying the electrochemical behavior of the enzymes, the surface coverage of GOD was 8.5 × 10^−10^ mol cm^−2^, and the electron transfer rate was 1.394 ± 0.02 s^−1^. The composite electrode showed good linearity for glucose in the range of 6.923 × 10^−2^ mM~1.54 mM, and the apparent Michaelis constant was 0.352 ± 0.02 mM. Furthermore, the GOD/chit^+^/PEDOT/AuDNs/EPLE sensor also achieved accurate glucose determination in human serum samples, although further modifications would be required for practical home applications. These findings demonstrated the potential of the AuDNs/EPLE composite electrode in the development of efficient enzymatic biosensors for glucose sensing applications.

## Figures and Tables

**Table 2:** Determination of glucose in real samples (n = 3).

Samples	Determined by Hospital (mM)	Our Measure (mM)			Average	RSD (%)
Sample 1	5.09	5.54	4.87	5.25	5.22	6.4
Sample 2	5.07	4.75	5.16	5.20	5.04	5.0
Sample 3	10.57	10.28	10.85	10.74	10.62	2.8
Sample 4	5.05	4.88	4.80	5.20	4.96	4.3
Sample 5	10.57	10.37	10.98	10.65	10.67	2.9

## Data Availability

The data presented in this study are available on request from the first author.
